# Engineering Enhanced Antimicrobial Properties in α-Conotoxin RgIA through D-Type Amino Acid Substitution and Incorporation of Lysine and Leucine Residues

**DOI:** 10.3390/molecules29051181

**Published:** 2024-03-06

**Authors:** Minghe Wang, Zhouyuji Liao, Dongting Zhangsun, Yong Wu, Sulan Luo

**Affiliations:** 1 Guangxi Key Laboratory of Special Biomedicine, School of Medicine, Guangxi University, Nanning 530004, China; 13793337608@163.com (M.W.); 15170245109@163.com (Z.L.); zhangsundt@163.com (D.Z.); 2 Key Laboratory of Tropical Biological Resources of Ministry of Education, Hainan University, Haikou 570228, China

**Keywords:** D-type amino acids, α-conotoxin, enzyme stability, safety

## Abstract

Antimicrobial peptides (AMPs), acknowledged as host defense peptides, constitute a category of predominant cationic peptides prevalent in diverse life forms. This study explored the antibacterial activity of α-conotoxin RgIA, and to enhance its stability and efficacy, D-amino acid substitution was employed, resulting in the synthesis of nine RgIA mutant analogs. Results revealed that several modified RgIA mutants displayed inhibitory efficacy against various pathogenic bacteria and fungi, including *Candida tropicalis* and *Escherichia coli*. Mechanistic investigations elucidated that these polypeptides achieved antibacterial effects through the disruption of bacterial cell membranes. The study further assessed the designed peptides’ hemolytic activity, cytotoxicity, and safety. Mutants with antibacterial activity exhibited lower hemolytic activity and cytotoxicity, with Pep 8 demonstrating favorable safety in mice. RgIA mutants incorporating D-amino acids exhibited notable stability and adaptability, sustaining antibacterial properties across diverse environmental conditions. This research underscores the potential of the peptide to advance innovative oral antibiotics, offering a novel approach to address bacterial infections.

## 1. Introduction

The middle of the 20th century was named the “antibiotic era” [1]. However, the overuse of antibiotics since the last century has led to drug-resistant microbial infections, which have become a significant health hazard for humans [2,3]. The development and introduction of new antimicrobial drugs have been significantly limited [4]. Therefore, the discovery of new antibiotics crucial for protecting human health is imperative [5,6]. Antimicrobial peptides (AMPs) have shown broad-spectrum antimicrobial activity and potent killing effects against a range of bacteria and fungi. Their ability to effectively combat drug-resistant pathogens has garnered significant attention [7]. However, to develop AMPs for medical use, several obstacles still need to be overcome, such as toxicity, stability, and bacterial resistance [8]. Many active AMPs comprise hydrophilic, hydrophobic, and cationic amino acids, forming amphiphilic structures that exert antimicrobial effects [9,10]. These amino acids, especially cationic amino acids such as arginine or lysine, are susceptible to trypsin degradation, which affects their stability in vivo, thus limiting the clinical application of antimicrobial peptides [11,12]. Therefore, several chemical modification strategies, such as peptidomimetic peptides, cyclization, and the use of amino acid analogs or non-natural amino acids, have been suggested to enhance the stability of AMPs.

In the natural milieu, amino acids manifest in two enantiomeric forms, L and D, except for glycine, which lacks a chiral center. The modification of AMPs through the incorporation of D-amino acids, evading recognition by human and microbial proteases, stands as a promising strategy to forestall premature hydrolytic degradation of proteins. This approach merits thorough exploration in the advancement of innovative AMP systems [13]. Peptides comprised of D-amino acids inherently display resistance to enzymatic protein hydrolysis, contrasting with their L-amino acid counterparts. Furthermore, concise linear or cyclic amphiphilic peptides, integrating both L- and D-amino acids, can impart variable degrees of selectivity and antimicrobial potency [14,15]. A prior investigation demonstrated that the integration of D-amino acids into a 12-amino-acid-long α-helical folding peptide preserved antimicrobial properties while attenuating hemolytic potential, despite the diminution of secondary structures in the resulting diastereomers [16].

Conotoxins are a large number of diverse biologically active peptides secreted by the venom glands of marine cones after a long evolution and are used for prey and defense. Conotoxins are mostly rich in disulfide bonds and act on a wide range of targets, such as ion channels, G protein-coupled receptors, transporters, and enzymes, making them highly attractive to neuroscientists and medicinal chemists [17]. Currently, several research groups have identified novel antimicrobial peptides in *Conus regius* or have undertaken modifications to enhance their antibacterial activity [18,19,20]. The α-conotoxin RgIA, originating from *Conus regius*, consists of a 13-residue short peptide. RgIA specifically binds to the α9α10 nicotinic acetylcholine receptor [21,22]. Owing to the presence of four positively charged arginine residues in its sequence, RgIA holds the potential for antimicrobial activity. In this study, we conducted an initial screening of the antibacterial activity of conotoxin RgIA. Subsequently, we introduced D-amino acids and designed nine mutant analogs of RgIA to assess antibacterial activity, stability, safety, and structural characteristics. This preliminary assessment contributes to establishing a foundation for enhancing the activity and stability of antimicrobial peptides through the introduction of D-type amino acids.

## 2. Results and Discussion

### 2.1. Synthesis of Antimicrobial Peptides

Based on the characteristics of antimicrobial peptides containing amphipathic amino acids, we used RgIA as a template for amino acid mutation. Specifically, we incorporated hydrophobic groups and positively charged groups by mutating glycine, serine, aspartate, proline, and tyrosine in the original sequence to lysine, arginine, and leucine amino acid residues. As the negatively charged components of the bacterial membrane serve as the first line of defense in the interaction with AMP, possessing a sufficient positive charge is a prerequisite for the effective functioning of the peptide. To investigate the impact of D-amino acids on peptide stability and activity, we incorporated D-lysine (D-Lys) and D-arginine (D-Arg) into select peptide chains due to their dual characteristics, namely, their possession of genuine positive charges and the fact that L-Arg and L-Lys serve as cleavage sites for several proteases, we substituted them with the corresponding D-amino acids. This substitution allows for the retention of their charge while preventing degradation by proteases, thereby enhancing stability. The specific sequences of the designed RgIA mutant peptides are presented in (Table 1). We utilized a two-step directed oxidation approach in the linkage of CysI-CysIII and CysII-CysIV. The first pair of disulfide bonds was formed through oxidation with potassium ferricyanide. After oxidizing CysI-CysIII with iodine, the resulting cyclic peptides underwent purification through RP-HPLC. Identification of the products was performed through analytical RP-HPLC and electrospray ionization–mass spectrometry (ESI-MS). All peptides were found to have a purity greater than 95%. The measured molecular weight of the synthetic peptide aligns with the theoretical molecular weight. RgIA analogs exhibit markedly heightened hydrophobicity when compared with the native form, potentially enhancing the antimicrobial activity of the peptides (Figure S1).

### 2.2. In Vitro Activity Assay

In the in vitro activity assay, we observed inhibitory effects of Pep 6 and Pep 8 on two fungi, as well as Pep 8’s activity against *Bacillus subtilis* and Pep 5’s activity against *Escherichia coli*. Notably, Pep 6 and Pep 8 were found to inhibit *Candida tropicalis* and *Candida parapsilosis* (Table 2).

**Figure 1:**
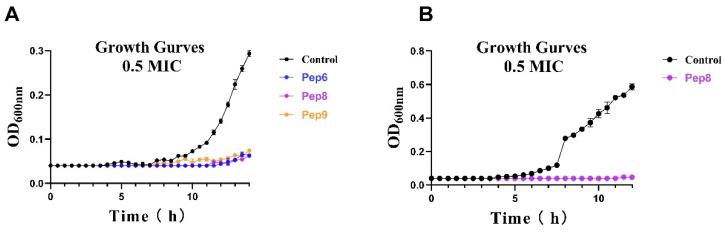
Growth curves in the presence and absence of antimicrobial peptides. (**A**) *Candida tropicalis.* (**B**) *Bacillus subtilis*.

**Figure 2:**
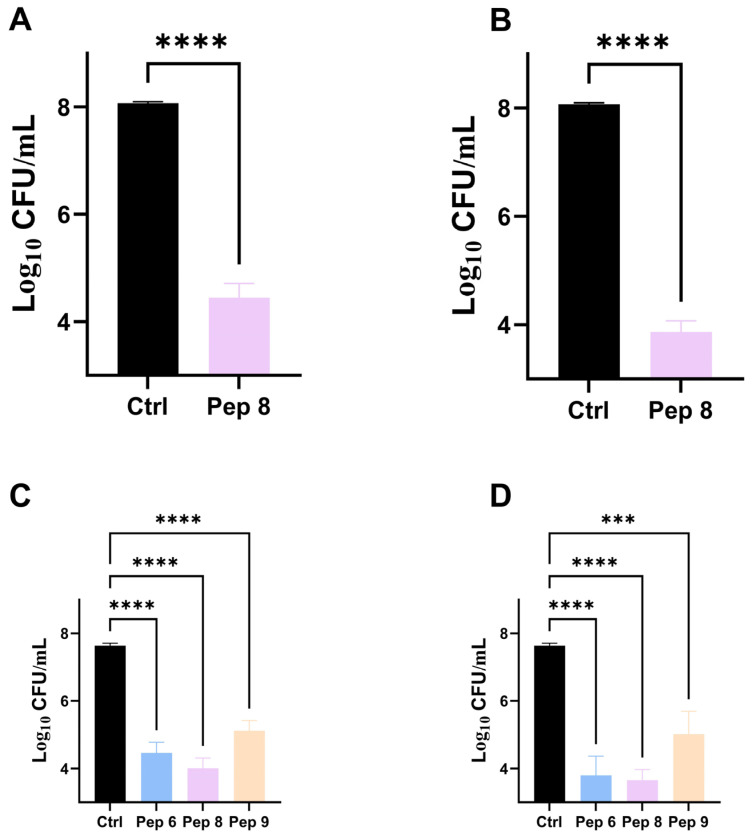
The bactericidal effect of 4 × MIC. (**A**) 30 min and (**B**) 60 min Pep 8 against Bacillus subtilis. The bactericidal effect of (**C**) 30 min and (**D**) 60 min Pep 6, Pep 8, Pep 9 against Candida tropicalis. ‘***’ indicates *p*-value less than 0.001 and ‘****’ indicates *p*-value less than 0.0001.

To comprehend the inhibitory impact of antimicrobial peptides on Bacteria and fungi at low concentrations, we plotted growth curves for *Bacillus subtilis* and *Candida tropicalis* and measured their OD values at 600 nm, as depicted in (Figure 1). Within 14 h, the control fungi exhibited significant growth measured at 600 nm with OD values ranging from 0.2 to 0.3. At the same time, we observed complete inhibition of the two fungi simply by adding 0.5 × MIC of antimicrobial peptides within the same 14 h period. Pep 6, Pep 8, and Pep 9 exhibited inhibitory effects on the growth of *Candida tropicalis*, while Pep 8 demonstrated inhibitory effects on the growth of *Bacillus subtilis*. To investigate further, we conducted time-killing kinetic experiments, as illustrated in (Figure 2). At four times the minimum inhibitory concentration, Pep 6, Pep 8 and Pep 9 were able to reduce fungal counts by over 1000-fold within 20 min. After 60 min, fungal counts dropped to 1 × 10^3^ cfu/mL, indicating a strong fungicidal effect. A common consensus in the scientific community is that if an antimicrobial peptide can reduce microbial counts by 1000-fold when compared with the initial state, it is considered bactericidal. If the number of microorganisms is reduced by less than 1000-fold from the initial state, then the antimicrobial peptide is considered to have only an inhibitory effect on the microorganisms [23]. The current experiments reveal that Pep 6 and Pep 8 display a significant bactericidal effect on *Candida tropicalis*. 

### 2.3. Stability

**Figure 3:**
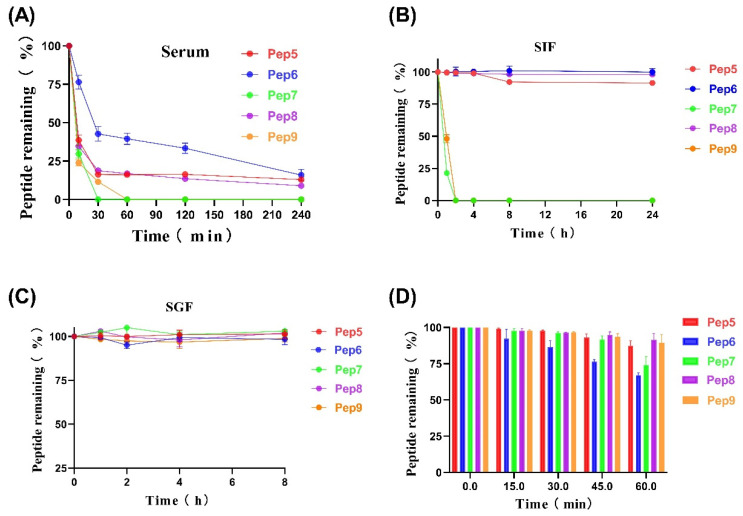
Stability of antimicrobial peptides. (**A**) Stability of antimicrobial peptides in 100% serum over 240 min. (**B**) Stability of antimicrobial peptides in simulated intestinal fluid over 24 h. (**C**) Stability of antimicrobial peptides in simulated gastric fluid over 8 h. (**D**) Thermal stability of antimicrobial peptides in a water bath at 80 °C over 60 min.

As shown in Figure 3A, Pep 5, Pep 6, and Pep 8, which incorporate D-type amino acids, have significantly better stability in the peptide serum when compared with Pep 7 and Pep 9, which lack D-type amino acids. The Pep 7 and Pep 9 were completely degraded within one hour, while the former still retained 20% stability after 4 h of incubation with 100% serum. 

The stability of the peptides in SIF is illustrated in Figure 3B. The result showed that Pep 5, Pep 6, and Pep 8 remained almost entirely intact after 24 h, while Pep 7 and Pep 9, which lacked D-amino acids, were nearly fully degraded within one hour. This could be attributable to trypsin preferentially degrading natural lysine and arginine residues, whereas the antimicrobial peptides we created feature a significant number of lysine and arginine residues. The manifested stability within SIF emphasizes its potential as an oral peptide pharmaceutical agent.

The stability of peptides in simulated gastric fluid (SGF) is illustrated in Figure 3C. All the peptides exhibited exceptional stability in this experiment, with minimal degradation within 8 h. This is attributed to the fact that pepsin primarily targets aromatic amino acids, which were not included in the design of the peptides to enhance stability and facilitate oral administration. This prevented the peptides from rapid degradation in SGF. Additionally, all synthesized peptides were highly stabilized when incubated at 80 °C for 1 h in the extreme temperature of the environment (Figure 3D). This stability helps protect the peptides from adverse effects that may occur during the application process. The robust stability exhibited by these antimicrobial peptides renders them potentially applicable in diverse production processes, including but not limited to food or feed processing and various other industrial domains.

### 2.4. CD Spectrum 

**Figure 4:**
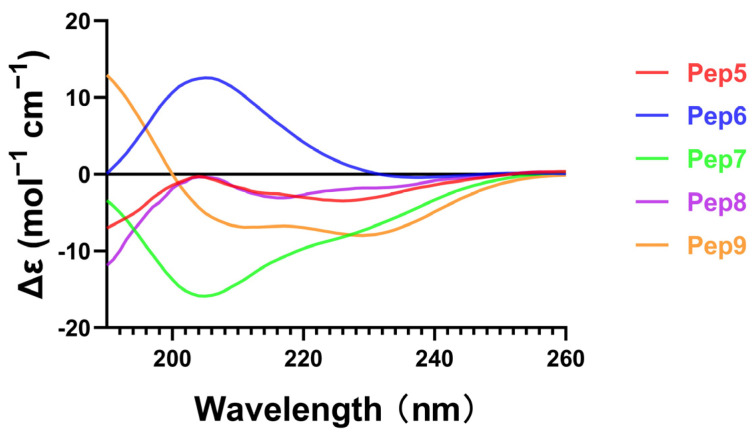
CD spectra of peptides.

The structure of antimicrobial peptides was analyzed using circular dichroism, as depicted in Figure 4. Generally, the α-helical structure of antimicrobial peptides plays a crucial role in their antimicrobial activity [24]. The α-helical structure of the peptide is characterized by two negative peaks at 208 and 222 nm. It is noticeable that Pep 9 exhibits a distinguishable α-helical configuration. On the other hand, this α-helical structure is absent in Pep 6, which has an identical sequence to Pep 9 but is changed by incorporating five D-type amino acids. The regularly arranged hydrogen bonds stabilize the helical structure. The incorporation of a significant number of d-amino acids into Pep 6 may have impacted the spatial localization of the cationic functional group, leading to a disruption of interactions between adjacent peptide molecules [13]. Despite the diastereomer’s loss of secondary structure, it managed to maintain its antimicrobial activity. The findings of this investigation demonstrate that the incorporation of D-amino acids can induce alterations in the secondary structure of AMPs. However, further specific research is needed to elucidate their influence on antibacterial activity.

### 2.5. Toxicity Test

Toxicity has been hindering the application and development of antimicrobial peptides due to concerns about hepatorenal toxicity and hemolytic effects. Our synthesized antimicrobial peptides, Pep 5 and Pep 6 containing D-type Lys, and Pep 8 containing D-type Arg, exhibited less than 5% hemolysis in hemolytic assays, even at a concentration of 128 μM (Table 3), indicating that these peptides containing D-type amino acids do not cause hemolytic toxicity.

**Figure 5:**
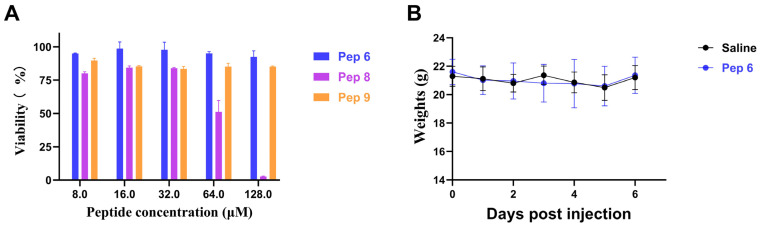
Safety Assessment of AMPs. (**A**) Cytotoxicity of antimicrobial Pep 6, 8, and 9. (**B**) In vivo safety of antimicrobial Pep 6 in C57BL/6 mice. Body weight changes in each group of mice (*n* = 5) treated with saline or 10 mg/kg of Pep 6 for 6 days.

Pep 6 and Pep 9 exhibited little or no toxicity on human hepatocyte THLE-3 at concentrations up to 128 μM, while Pep 8, which contains a higher amount of D-type Arg, displayed remarkable cytotoxicity at both 64 μM and 128 μM (Figure 5A). It is worth noting that Pep 8’s cytotoxicity increased with its concentration. It is possible that Arg-containing peptides have more potent electrostatic interactions than lysine-rich peptides, while R can pull lipid phosphate groups, causing impaired ring pores in cell membrane lumens. While our synthesized Pep 8 exhibited commendable antibacterial properties, it also presented a degree of cytotoxicity at higher concentrations, indicating the need for additional optimization.

In toxicity experiments on mice, we discovered that Pep 6 is exceptionally biologically secure. We demonstrated this by administering a daily injection of Pep 6 at a dosage of 10 mg/kg to C57BL/6 mice for seven days (Figure 5B). There was a slight decrease in body weight of the treated mice, approximately 1 g on the first day, with no significant changes in the following six days.

### 2.6. Detection of Antimicrobial Mechanisms

The current experimental findings elucidate that the principal mechanism underlying antimicrobial activity involves the disruption of microbial cell membranes. Laser confocal microscopy facilitated fluorescence imaging, while scanning electron microscopy was employed for the assessment of cell membrane disruption.

**Figure 6:**
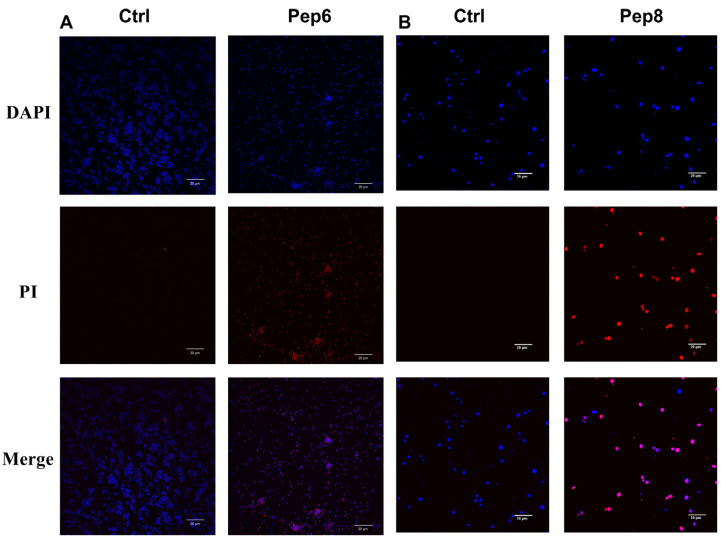
Fluorescence confocal microscopic images of (**A**) *Bacillus subtilis* or (**B**) *Candida tropicalis* after treatment with Pep 6 or 8 (4 × MIC) for 30 min.

Laser confocal microscopy was applied for the observation of bacterial fluorescence, employing DAPI and PI. DAPI, capable of penetrating the cell membrane, adheres to the DNA double helix structure, whereas PI is incapable of traversing intact cell membranes. Consequently, the red fluorescence effect was not discernible in the treated live cells. Blue fluorescence was observed in both the sample groups treated with antimicrobial peptides and the control group treated with PBS (10 mM) (Figure 6). Red fluorescence was exclusively observed in cells treated with antimicrobial peptides.

**Figure 7:**
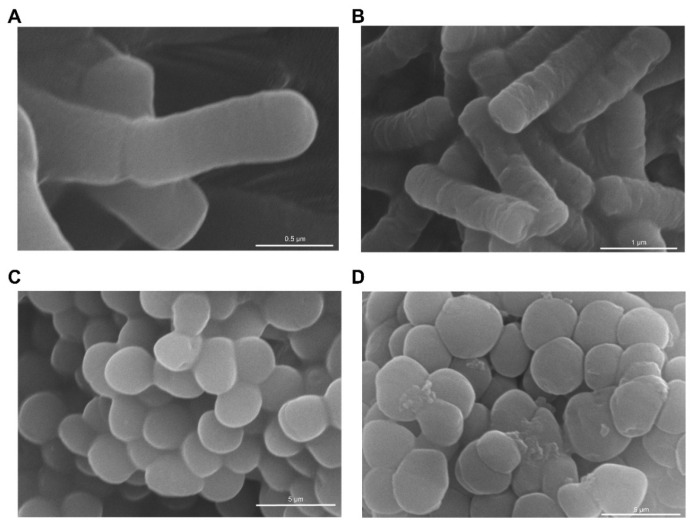
SEM images of (**A**) *Bacillus subtilis* or (**C**) *Candida tropicalis* without the addition of antimicrobial peptides. SEM images of (**B**) *Bacillus subtilis* or (**D**) *Candida tropicalis* after treatment with Pep 6 or 8 (4 × MIC) for 30 min.

The cellular morphology of *Bacillus subtilis* and *Candida tropicalis*, as observed under a scanning electron microscope, is delineated in (Figure 7). Cells subjected to PBS buffer (10 mM) exhibited an intact surface with no discernible rupture, while organisms treated with the antimicrobial peptide displayed varying degrees of surface rupture. We postulate that physical membrane damage constitutes the primary causative factor for cell death. Our investigation indicates that our antimicrobial peptides proficiently penetrate and disrupt bacterial membranes, leading to expeditious bactericidal effects with limited potential for the development of drug resistance. The consistent findings across our research suggest that the predominant mechanism of action for most AMPs involves targeting and disrupting the lipid bilayer structure of bacterial membranes.

## 3. Experimental Section

### 3.1. Materials

The materials utilized in this study including N,N-Dimethylformamide (DMF), Dichloromethane (DCM), ether, hydrochloric acid, 4′,6-diamidino-2-phenylindole (DAPI), and propidium iodide (PI) were purchased from Aladdin (Shanghai China); The CCK-8 kit was purchased from Sampo Bio (Shanghai, China); Glutaraldehyde (2.5%) was purchased from Sevier (Wuhan China); Trypsin (250 U/mg) and pepsin (>3000 U/mg) were purchased from McLean (Shanghai China); Hepatic THLE-3 cells were kindly donated by Dr. Shieng Chen from Guangxi University. *Candida tropicalis* (BNCC340288), *Candida parapsilosis* (BNCC336015), *Bacillus subtilis* (BNCC109047), and *Escherichia coli* (BNCC336902) strains used in the experiment were purchased from Beina Biotechnology (Hebei, China). All materials were of high purity and underwent no further purification.

### 3.2. Synthesis of Antimicrobial Peptides

Initially, conotoxin linear peptides were synthesized using a microwave peptide synthesizer (CEM Liberty Blue America), with the oxidative folding following a protocol consistent with our previous studies [25,26,27]. To form two correct disulfides (CysI-CysIII, CysII-CysIV), we utilized a combination of trityl (Trt) and acetamidomethyl (Acm) for side chain protection of cysteines. Specifically, resin cleavage was conducted at 40 °C using trifluoroacetic acid, H_2_O, 3,6-dioxa-1,8-octanedithiol (DODT), and triisopropylsilane, with a ratio of 92.5:2.5:2.5:2.5. After precipitation with ether and centrifugal drying, the product underwent preparative reverse-phase–high-performance liquid chromatography (RP-HPLC) (Waters Prep 150 LC system America) purification, reaching a purity exceeding 90%. By employing a two-step oxidation approach, two pairs of disulfide bonds were formed in the linear peptide. The collected peptides were then diluted with water for the first part of oxidation. Potassium ferrocyanide (20 mM) and Tris (0.1 M) oxidized the linear peptide solutions for 40 min at room temperature, yielding the initial CysI-CysIII disulfide bonds. The product then underwent a subsequent round of preparative RP-HPLC. Products with purity exceeding 90% proceeded to the second oxidation step. Iodine oxidation removed the Acm protective group on cysteine. The resulting RgIA analog was confirmed for its theoretical molecular weight using electrospray mass spectrometry. The final product’s purity was assessed via analytical RP-HPLC (Waters Alliance e2695 America), yielding peptides with potential antimicrobial activity with a purity surpassing 98%, ready for further characterization.

### 3.3. Antimicrobial Peptide Stability Analysis

In accordance with the methodology employed in the study by Zhao, J. et al. we examined the thermal stability of antimicrobial peptides through incubation in hot water [20,28]. In summary, 100 nmol of the peptide was dissolved in 1 mL of distilled water, then incubated at 80 °C. At 0, 15, 30, 45, and 60 min, 50 μL of the peptide was taken and cooled in room temperature water. RP-UPLC was used to detect the peak area of each sample, with the 0 min peak area serving as the baseline at 100%. This allowed us to calculate the amount of peptide remaining at various time points. 

The serum stability is referenced from the study by He, T. et al. [29]. The serum stability of antimicrobial peptides was measured using cryopreserved serum, which was thawed in a 4 °C refrigerator for 12 h prior to incubation at 37 °C for 15 min. We added 1 mL of serum to 100 nmol of antimicrobial peptide and incubated it at 37 °C. We extracted 30 μL of the antimicrobial peptide serum solution and mixed it with 90 μL of 12% trichloroacetic acid to halt the reaction at 0, 30, 60, 120, and 240 min, respectively. RP-UPLC was utilized to determine the peak area after centrifugation.

Stability analysis of synthetic intestinal fluid (SIF) containing antimicrobial peptides was carried out by following the previous study by Jie, R. et al. [30,31,32]. To prepare the artificial intestinal fluid, 100 mg of trypsin was added to 10 mL of water, and the pH was adjusted to 6.8 using potassium dihydrogen phosphate and sodium hydroxide. A total of 100 mmol of antimicrobial peptides was dissolved in 1 mL of artificial intestinal solution. The solution was then incubated in a water bath at 37 °C. After that, 50 mL of the antimicrobial solution was obtained and mixed with 50 microliters of 4% trifluoroacetic acid to stop the reaction at 0, 1, 2, 4, 8, and 24 h, respectively. RP-UPLC was used to detect the peak area of the samples at each time point.

For the stability analysis of the antimicrobial peptide in the artificial gastric fluid (SGF), we mainly employed the method reported by Jakobek et al. [33]. In short, we added 1 mg of pepsin to 100 mL of water and adjusted the pH to 1.5 with HCl to obtain the artificial gastric fluid. Next, we dissolved 100 nmol of the antimicrobial peptide in SGF and incubated it at 37 °C. The reaction was stopped by transferring 50 μL to Na_2_CO_3_ solutions (0.2 M) at 0, 1, 2, 4, and 8 h time points. The peak area was measured using RP-UPLC. All stability experiments for the antimicrobial peptides were repeated three times.

### 3.4. Determination of Minimum Inhibitory Concentration (MIC) of Antimicrobial Peptides 

MIC of Antimicrobial Peptides is crucial for effective treatment. The bacteria were incubated at 37 °C for 24 h, while the fungi were incubated at 28 °C for 24 h. Bacterial colonies in all solutions were diluted to OD_600_ = 0.4 and then the concentration was reduced by 1000 times and prepared for use [34]. The MIC was then calculated. To each well of a 96-well plate, 50 μL of peptide (8 μM–128 μM) solutions were added. A diluted bacterial solution (1 × 10^5^ cfu/mL) was added to each well, followed by incubation of the 96-well plates at 37 °C and 28 °C for 24 h in an incubator. A microplate reader (SpectraM axM2/MD America) at OD_600_ was utilized to determine the lowest inhibitory concentration of the antimicrobial peptide [35]. For each sample, three replicate wells were established, and the experiment was repeated thrice.

### 3.5. Growth Curves and Bactericidal Effects 

The growth inhibition curves of antimicrobial peptides against two strains of bacteria were established using Li Wang’s method [36]. Bacterial concentrations were diluted to (1 × 10^5^ cfu/mL) as indicated in the MIC determination of antimicrobial peptides protocol. For the 96-well plate, 50 μL of 10 mM phosphate-buffered saline (PBS, pH 7.4) buffer was added to each well, and 50 μL of bacterial solution was added as a control. Then 50 μL of antimicrobial peptide at a concentration of 1 × MIC with 50 μL of diluted bacterial and fungal solution was added to a 96-well plate to give a final concentration of 0.5 × MIC of the peptide. The 96-well plate was subsequently incubated at a constant temperature and removed every thirty minutes to determine the OD_600_ using a microplate reader assay. This process was repeated twenty-eight times. For both control and sample groups, three replicate wells were created at a time, each repeated three times.

The bactericidal effect was assessed using previously established methods [37,38]. The two bacterial strains were diluted to a final concentration of approximately 1 × 10^8^ cfu/mL. Next, 250 μL of the bacterial solution and 250 μL of 4 × MIC peptide solution were combined in a centrifuge tube and incubated at 28 ℃ for 15 min. Afterward, 50 μL of the mixture was evenly spread on an agar plate, and the number of colonies was determined after incubation in a temperature-controlled incubator for 18–24 h. A control group consisting of a strain sample with PBS (10 mM) added was utilized.

### 3.6. CD Spectra

In order to delineate the secondary structural attributes of the altered peptides, CD spectroscopy was employed for the examination of the synthesized peptides. Specifically, antimicrobial peptides with a final concentration of 100 μM were tested using CD spectroscopy (λ 190–260 nm), and the recorded CD spectra were then converted to mean residue ellipticity using the following equation.

*θ*_M_ = (*θ*_obs_ × 1000)/*c* ln (1)

In this equation, *θ*_M_ represents the residual ellipticity in degrees per square centimeter per mole, *θ*_obs_ represents the observed ellipticity corrected for the buffer at a given wavelength in millidegrees, c represents the peptide concentration (in mM), l represents the path length (in mm), and n represents the number of amino acid residues.

### 3.7. Hemolytic Assay 

The hemolysis assay procedures are consistent with the previous study by Ngambenjawong et al. [39]. Blood samples were collected from healthy C57BL/6 mice. The collected whole blood was then placed in blood collection tubes containing anticoagulants and left for an hour. Afterward, the supernatant was centrifuged, and the resulting hemoglobin cells were washed three times with PBS buffer (10 mM, pH 7.4). The hemoglobin cells were then diluted to 10% concentration and kept on standby. The antimicrobial peptide concentration was sequentially diluted to 512, 256, 128, 64, 32, and 16 μM. Next, 50 μL of the resultant solution was mixed with 150 μL of 10% hemoglobin cells, followed by incubation at 37 °C for 1 h. The supernatant was then centrifuged and transferred to a 96-well plate. Finally, the absorbance value was measured using a microplate reader at OD_540_ [40]. The study included a positive control with 0.1% trilactone and a negative control with PBS buffer (10 mM, pH 7.4) instead of antimicrobial peptide. The percentage of hemolysis was determined using the following formula:

Hemolysis percentage = [(*A* − *A*_0_)/(*A_T_* − *A*_0_)] × 100%. (2)

In this formula, *A*, *A*_0_, and *A_T_* represent the absorbance values of the antimicrobial peptide at 570 nm, negative control, and positive control, respectively. Three sets of parallel experiments were arranged for every concentration of antimicrobial peptide in the hemolytic assay and repeated three times.

### 3.8. Cytotoxicity 

Cytotoxicity was assessed using human hepatocytes (THLE-3) and the CCK-8 kit, following a modified version of a previously reported method [24,41]. The methodology employed was as follows: Using a 96-well plate, cells were cultured to a density of around 5000 cells per well. The antimicrobial peptide was then added to the plates to reach final concentrations of 128, 64, 32, 16, and 8 μM, and were subsequently incubated for 36 h at 37 °C in a cell culture incubator with 5% CO_2_. Following this, 10 μL of CCK-8 reagent was added to each well, and the OD values were measured at 490 nm after the plates were incubated at 37 °C for an additional two hours. Cell viability was calculated using the formula:

Survival rate = [(OD of sample cells − OD of blank)/(OD of control cells − OD of blank)] × 100(3)

Those with peptide and CCK-8 reagent added were the sample group, those with CCK-8 reagent added and no peptide added were the control group, and those without antimicrobial peptide and CCK-8 reagent added were the blank group. Cell viability measurements were timed to ensure that there were three replicates per sample point and three repeats in total.

### 3.9. Fluorescence Imaging by Laser Confocal Microscopy

Fluorescence imaging through laser confocal microscopy was conducted following the procedure by Hisham, R.I. et al. [42,43]. Antimicrobial peptides were added to suspensions of *Candida tropicalis* and *Bacillus subtilis* (at a concentration of approximately 1 × 10^7^ cfu/mL) in a volume equal to that of the bacterial and fungal fluids, at a concentration four times the minimum inhibitory concentration (4 × MIC). The suspension was then incubated for one hour. The precipitate was subsequently gathered by centrifugation and washed thrice with 10 mM PBS (pH 7.4) to maintain the bacteria’s suspension state in the PBS buffer (10 mM). Then 20 μL of PI (10 μg/mL) and 20 μL of DAPI (μg/mL) were added to a 100 μL bacterial suspension, and the mixture was incubated at 4 °C, away from light, for 30 min before transferring 10 μL onto slides. The fluorescence effect was observed using a laser confocal microscope (Olympus FV3000 America).

### 3.10. Scanning Electron Microscopy

Scanning electron microscopy was performed by incubating colonies to OD_600_ = 0.4, co-incubating them with the same volume of antimicrobial peptide (4 × MIC) for 1 h. The colonies were then rinsed three times with pH 7.4 PBS buffer (10 mM), centrifuged at 3500 rpm for 5 min, and fixed overnight with 2.5% (*w*/*v*) glutaraldehyde electron microscopy fixative. Subsequently, the colonies underwent dehydration and drying utilizing varying concentrations of ethanol (30%, 50%, 70%, 90%, and 100%). After the ethanol removal, the colonies were dried for one day and then coated with gold. A swept electron microscope (HITACHI SU5000 Japan) was utilized to observe the effect of bacterial breakage [44,45].

### 3.11. In Vivo Toxicity Analysis

In vivo toxicity analysis was carried out using twelve healthy adult C57BL/6 mice divided into two groups. The sample group received daily intraperitoneal injections of 10 mg/kg antimicrobial peptide, while the control group received an equal volume of saline injections. Daily measurements of the mice’s weight were taken for six consecutive days [46].

### 3.12. Statistical Analysis 

All experimental data were repeated at least three times. Experimental data were analyzed with GraphPad Prism 8 and expressed as mean ± SD. One-way ANOVA and *t* tests were used to compare variables, and data with *p* < 0.05 were deemed statistically significant.

## 4. Conclusions

In this study, analogs of the D-amino acid α-conotoxin RgIA were synthesized and tested for antimicrobial properties and stability. Pep 5, 6, and 8 displayed high levels of stability in 100% serum, SIF, and SGF, as well as in a water bath at 80 °C. Pep 6 and 8 demonstrated excellent bactericidal effects, while Pep 6 exhibited minimal adverse effects on hemoglobin and hepatocytes at a concentration of 128 μM. The safety of Pep 6 was also affirmed in vivo toxicity experiments using mice. Our designed antimicrobial Pep 5 and 6, which contain D-type amino acids, exhibit a significantly enhanced bactericidal effect when compared with antimicrobial Pep 7 and 9, which have the same sequence but utilize L-type amino acids. Moreover, their stability has been remarkably improved, and they exhibit strong resistance to external environmental influences. 

## Figures and Tables

**Table 1:** Physicochemical Properties of the Designed Peptides.

Peptides	Sequences	AA	Theoretical MW (Da)	Measured MW (Da)	Net Charge
RgIA	GC^I^C^II^SDPrC^III^rYrC^IV^#	12	1417.66	1416.27	+3
Pep 1	GC^I^C^II^LkLkC^III^kYkC^IV^#	12	1388.83	1387.44	+4
Pep 2	LC^I^C^II^LkLkC^III^kWkC^IV^#	12	1467.98	1466.57	+4
Pep 3	LC^I^C^II^LkLkC^III^kLkC^IV^#	12	1394.92	1393.59	+4
Pep 4	LC^I^C^II^LkLkAC^III^kLkC^IV^#	13	1466.00	1464.48	+4
Pep 5	LC^I^C^II^LkC^III^kLkLkC^IV^#	12	1394.92	1393.59	+4
Pep 6	LkkLC^I^C^II^kC^III^kLkC^IV^#	12	1409.94	1408.05	+5
Pep 7	LC^I^C^II^LKC^III^KLKLKC^IV^#	12	1394.92	1392.99	+4
Pep 8	LC^I^C^II^LrC^III^rLkLrC^IV^#	12	1478.97	1477.41	+4
Pep 9	LKKLC^I^C^II^KC^III^KLKC^IV^#	12	1409.94	1408.53	+5

#C-terminal amidation (NH2). AA: number of amino acid residues contained. Measured MW was tested by mass spectrometry (MS). The disulfide bond connections in the sequences were all CysI–CysIII and CysII–CysIV. Lowercase letters represent the corresponding D-amino acids. Roman numerals I–IV represent disulfide bond positions in the sequence.

**Table 2:** MICs (μM) of AMPs.

Peptides	*C. tropical*	*C. parapsilosis*	*B. subtilis*	*E. coli*
RgIA	128	>128	>128	>128
Pep 1	>64	>64	>64	>64
Pep 2	>64	>64	>64	>64
Pep 3	>64	>64	>64	>64
Pep 4	>64	>64	>64	>64
Pep 5	64	128	128	32
Pep 6	8	32	64	128
Pep 7	64	64	64	>128
Pep 8	8	8	16	128
Pep 9	16	16	128	>128
clotrimazole	12.5	25	-	-

**Table 3:** Hemolytic toxicity of antimicrobial Peptides.

Erythrocyte Lysis (%)							
Peptides (μM)	2	4	8	16	32	64	128
Pep 5	0.37	−0.03	−0.19	−0.17	−0.96	−0.49	0.32
Pep 8	0.24	0.18	0.35	0.18	0.94	1.17	0
Pep 9	0.54	0.01	1.16	0.43	−0.58	−0.67	0.01

## Data Availability

Data are contained within the article and Supplementary Materials.

## Supplementary Materials

The following supporting information can be downloaded at: https://www.mdpi.com/article/10.3390/molecules29051181/s1, Figure S1: (a) LC-MS analysis of Pep 1. (b) HPLC analysis of the Pep 1. (c) LC-MS analysis of Pep 2. (d) HPLC analysis of the Pep 2. (e) LC-MS analysis of Pep 3. (f) HPLC analysis of the Pep 3. (g) LC-MS analysis of Pep 4. (h) HPLC analysis of the Pep 4. (i) LC-MS analysis of Pep 5. (j) HPLC analysis of the Pep 5. (k) LC-MS analysis of Pep 6. (l) HPLC analysis of the Pep 6. (m) LC-MS analysis of Pep 7. (n) HPLC analysis of the Pep 7. (o) LC-MS analysis of Pep 8. (p) HPLC analysis of the Pep 8. (q) LC-MS analysis of Pep 9. (r) HPLC analysis of the Pep 9.

## References

[1] Huemer M., Mairpady Shambat S., Brugger S.D., Zinkernagel A.S. (2020). Antibiotic resistance and persistence-Implications for human health and treatment perspectives. EMBO Rep..

[2] Agerberth B., Lee J.Y., Bergman T., Carlquist M., Boman H.G., Mutt V., Jornvall H. (1991). Amino acid sequence of PR-39. Isolation from pig intestine of a new member of the family of proline-arginine-rich antibacterial peptides. Eur. J. Biochem..

[3] Marston H.D., Dixon D.M., Knisely J.M., Palmore T.N., Fauci A.S. (2016). Antimicrobial Resistance. JAMA.

[4] Lewis K. (2013). Platforms for antibiotic discovery. Nat. Rev. Drug Discov..

[5] Chou S., Shao C., Wang J., Shan A., Xu L., Dong N., Li Z. (2016). Short, multiple-stranded β-hairpin peptides have antimicrobial potency with high selectivity and salt resistance. Acta Biomater..

[6] Zasloff M. (2002). Antimicrobial peptides of multicellular organisms. Nature.

[7] Magana M., Pushpanathan M., Santos A.L., Leanse L., Fernandez M., Ioannidis A., Giulianotti M.A., Apidianakis Y., Bradfute S., Ferguson A.L. (2020). The value of antimicrobial peptides in the age of resistance. Lancet Infect. Dis..

[8] Shao C., Zhu Y., Jian Q., Lai Z., Tan P., Li G., Shan A. (2021). Cross-Strand Interaction, Central Bending, and Sequence Pattern Act as Biomodulators of Simplified β-Hairpin Antimicrobial Amphiphiles. Small.

[9] Willyard C. (2017). Drug-resistant bacteria ranked. Nature.

[10] Zhu X., Zhang L., Wang J., Ma Z., Xu W., Li J., Shan A. (2015). Characterization of antimicrobial activity and mechanisms of low amphipathic peptides with different α-helical propensity. Acta Biomater..

[11] Kim H., Jang J.H., Kim S.C., Cho J.H. (2014). De novo generation of short antimicrobial peptides with enhanced stability and cell specificity. J. Antimicrob. Chemother..

[12] Zhu Y., Shao C., Li G., Lai Z., Tan P., Jian Q., Cheng B., Shan A. (2020). Rational Avoidance of Protease Cleavage Sites and Symmetrical End-Tagging Significantly Enhances the Stability and Therapeutic Potential of Antimicrobial Peptides. J. Med. Chem..

[13] Ong Z.Y., Cheng J., Huang Y., Xu K., Ji Z., Fan W., Yang Y.Y. (2014). Effect of stereochemistry, chain length and sequence pattern on antimicrobial properties of short synthetic β-sheet forming peptide amphiphiles. Biomaterials.

[14] Fernandez-Lopez S., Kim H.S., Choi E.C., Delgado M., Granja J.R., Khasanov A., Kraehenbuehl K., Long G., Weinberger D.A., Wilcoxen K.M. (2001). Antibacterial agents based on the cyclic D,L-α-peptide architecture. Nature.

[15] Maloy W.L., Kari U.P. (1995). Structure-activity studies on magainins and other host defense peptides. Biopolymers.

[16] Oren Z., Hong J., Shai Y. (1997). A repertoire of novel antibacterial diastereomeric peptides with selective cytolytic activity. J. Biol. Chem..

[17] Jin A.-H., Muttenthaler M., Dutertre S., Himaya S.W.A., Kaas Q., Craik D.J., Lewis R.J., Alewood P.F. (2019). Conotoxins: Chemistry and Biology. Chem. Rev..

[18] Bernaldez-Sarabia J., Figueroa-Montiel A., Duenas S., Cervantes-Luevano K., Beltran J.A., Ortiz E., Jimenez S., Possani L.D., Paniagua-Solis J.F., Gonzalez-Canudas J. (2019). The Diversified O-Superfamily in Californiconus californicus Presents a Conotoxin with Antimycobacterial Activity. Toxins.

[19] Figueroa-Montiel A., Bernaldez J., Jimenez S., Ueberhide B., Javier Gonzalez L., Licea-Navarro A. (2018). Antimycobacterial Activity: A New Pharmacological Target for Conotoxins Found in the First Reported Conotoxin from Conasprella ximenes. Toxins.

[20] Hemu X., Tam J.P. (2017). Macrocyclic Antimicrobial Peptides Engineered from ω-Conotoxin. Curr. Pharm. Des..

[21] Ellison M., Haberlandt C., Gomez-Casati M.E., Watkins M., Elgoyhen A.B., McIntosh J.M., Olivera B.M. (2006). α-RgIA: A novel conotoxin that specifically and potently blocks the α9α10 nAChR. Biochemistry.

[22] Mannelli L.D.C., Cinci L., Micheli L., Zanardelli M., Pacini A., McIntosh J.M., Ghelardini C. (2014). α-Conotoxin RgIA protects against the development of nerve injury-induced chronic pain and prevents both neuronal and glial derangement. Pain.

[23] Kalia V., Miglani R., Purnapatre K.P., Mathur T., Singhal S., Khan S., Voleti S.R., Upadhyay D.J., Saini K.S., Rattan A. (2009). Mode of Action of Ranbezolid against Staphylococci and Structural Modeling Studies of Its Interaction with Ribosomes. Antimicrob. Agents Chemother..

[24] Lai Z., Tan P., Zhu Y., Shao C., Shan A., Li L. (2019). Highly Stabilized Amphiphiles Coiled Coils Kill Gram-Negative Bacteria by Multicomplementary Mechanisms under Acidic Condition. ACS. Appl. Mater. Interfaces.

[25] Luo S., Zhangsun D., Zhu X., Wu Y., Hu Y., Christensen S., Harvey P.J., Akcan M., Craik D.J., McIntosh J.M. (2013). Characterization of a Novel α-Conotoxin TxID from Conus textile That Potently Blocks Rat α3β4 Nicotinic Acetylcholine Receptors. J. Med. Chem..

[26] Wu X., Wu Y., Zhu F., Yang Q., Wu Q., Zhangsun D., Luo S. (2013). Optimal Cleavage and Oxidative Folding of α-Conotoxin TxIB as a Therapeutic Candidate Peptide. Mar. Drugs.

[27] Zhu X., Pan S., Xu M., Zhang L., Yu J., Yu J., Wu Y., Fan Y., Li H., Kasheverov I.E. (2020). High Selectivity of an α-Conotoxin LvIA Analogue for α3β2 Nicotinic Acetylcholine Receptors Is Mediated by β2 Functionally Important Residues. J. Med. Chem..

[28] Zhao J., Fan R., Jia F., Huang Y., Huang Z., Hou Y., Hu S.-Q. (2021). Enzymatic Properties of Recombinant Ligase Butelase-1 and Its Application in Cyclizing Food-Derived Angiotensin I-Converting Enzyme Inhibitory Peptides. J. Agric. Food Chem..

[29] He T., Xu L., Hu Y., Tang X., Qu R., Zhao X., Bai H., Li L., Chen W., Luo G. (2022). Lysine-Tethered Stable Bicyclic Cationic Antimicrobial Peptide Combats Bacterial Infection In Vivo. J. Med. Chem..

[30] Emidio N.B., Tran H.N.T., Andersson A., Dawson P.E., Albericio F., Vetter I., Muttenthaler M. (2021). Improving the Gastrointestinal Stability of Linaclotide. J. Med. Chem..

[31] Ren J., Zhu X., Xu P., Li R., Fu Y., Dong S., Zhangsun D., Wu Y., Luo S. (2019). D-Amino Acid Substitution of α-Conotoxin RgIA Identifies its Critical Residues and Improves the Enzymatic Stability. Mar. Drugs.

[32] Zhang Y., Cao J., Wang X., Liu H., Shao Y., Chu C., Xue F., Bai J. (2022). The effect of enzymes on the in vitro degradation behavior of Mg alloy wires in simulated gastric fluid and intestinal fluid. Bioact. Mater..

[33] Jakobek L., Strelec I., Kenjeric D., Soher L., Tomac I., Matic P. (2022). Simulated Gastric and Intestinal Fluid Electrolyte Solutions as an Environment for the Adsorption of Apple Polyphenols onto β-Glucan. Molecules.

[34] Li R., Wang X., Yin K., Xu Q., Ren S., Wang X., Wang Z., Yi Y. (2023). Fatty acid modification of antimicrobial peptide CGA-N9 and the combats against Candida albicans infection. Biochem. Pharmacol..

[35] Ali Mohammadie Kojour M., Edosa T.T., Jang H.A., Keshavarz M., Jo Y.H., Han Y.S. (2021). Critical Roles of Spatzle5 in Antimicrobial Peptide Production Against Escherichia coli in Tenebrio molitor Malpighian Tubules. Front. Immunol..

[36] Wang L., Liu L., Wang X., Tan Y., Duan X., Zhang C., Cheng J., Xiong Y., Jiang G., Wang J. (2022). Ruthenium(II) complexes targeting membrane as biofilm disruptors and resistance breakers in Staphylococcus aureus bacteria. Eur. J. Med. Chem..

[37] Mwangi J., Yin Y., Wang G., Yang M., Li Y., Zhang Z., Lai R. (2019). The antimicrobial peptide ZY4 combats multidrug-resistant Pseudomonas aeruginosa and baumannii infection. Proc. Natl. Acad. Sci. USA.

[38] Wang Y., Zhang Z., Chen L., Guang H., Li Z., Yang H., Li J., You D., Yu H., Lai R. (2011). Cathelicidin-BF, a snake cathelicidin-derived antimicrobial peptide, could be an excellent therapeutic agent for acne vulgaris. PLoS ONE.

[39] Ngambenjawong C., Chan L.W., Fleming H.E., Bhatia S.N. (2022). Conditional Antimicrobial Peptide Therapeutics. ACS Nano.

[40] Locock K.E.S., Michl T.D., Valentin J.D.P., Vasilev K., Hayball J.D., Qu Y., Traven A., Griesser H.J., Meagher L., Haeussler M. (2013). Guanylated Polymethacrylates: A Class of Potent Antimicrobial Polymers with Low Hemolytic Activity. Biomacromolecules.

[41] Jin L., Bai X., Luan N., Yao H., Zhang Z., Liu W., Chen Y., Yan X., Rong M., Lai R. (2016). A Designed Tryptophan- and Lysine/Arginine-Rich Antimicrobial Peptide with Therapeutic Potential for Clinical Antibiotic-Resistant Candida albicans Vaginitis. J. Med. Chem..

[42] Ibrahim H.R., Imazato K., Ono H. (2011). Human Lysozyme Possesses Novel Antimicrobial Peptides within Its N-terminal Domain that Target Bacterial Respiration. J. Agric. Food Chem..

[43] Mochon A.B., Liu H. (2008). The Antimicrobial Peptide Histatin-5 Causes a Spatially Restricted Disruption on the Candida albicans Surface, Allowing Rapid Entry of the Peptide into the Cytoplasm. PLoS Pathog..

[44] Miao F., Tai Z., Wang Y., Zhu Q., Fang J.K.-H., Hu M. (2022). Tachyplesin I Analogue Peptide as an Effective Antimicrobial Agent against Candida albicans Staphylococcus aureus Poly-Biofilm Formation and Mixed Infection. ACS Infect. Dis..

[45] Wei L., Gao J., Zhang S., Wu S., Xie Z., Ling G., Kuang Y.-Q., Yang Y., Yu H., Wang Y. (2015). Identification and Characterization of the First Cathelicidin from Sea Snakes with Potent Antimicrobial and Anti-inflammatory Activity and Special Mechanism. J. Biol. Chem..

[46] Mishra B., Narayana J.L., Lushnikova T., Wang X., Wang G. (2019). Low cationicity is important for systemic in vivo efficacy of database-derived peptides against drug-resistant Gram-positive pathogens. Proc. Natl. Acad. Sci. USA.

