# Supplementary material for: Engineering Enhanced Antimicrobial Properties in α-Conotoxin RgIA through D-Type Amino Acid Substitution and Incorporation of Lysine and Leucine Residues
Source: Molecules. 2024 Mar 6;29(5):1181. doi: 10.3390/molecules29051181 (PMC10935098; doi:10.3390/molecules29051181)
Supplement: Supplementary file 1 [file molecules-29-01181-s001.zip › molecules-2852514-supplementary.pdf]

Supporting Information

Designing Antimicrobial Peptides Based on α-Conotoxin RgIA

Minghe Wang [1], Zhouyuji Liao [1], Dongting Zhangsun [1,2], Yong Wu [1,]* and Sulan Luo [1,2,]*

[1] School of Medicine, Guangxi University, Nanning 530004, China; 13793337608@163.com (M.W.); 15170245109@163.com (Z.L.); zhangsundt@163.com (D.Z.)
[2] Key Laboratory of Tropical Biological Resources of Ministry of Education, Hainan University, Haikou 570228, China
* Correspondence: wuyong@gxu.edu.cn (Y.W.); sulan2021@gxu.edu.cn (S.L.)

LC-MS and HPLC analysis of peptides.

(a)

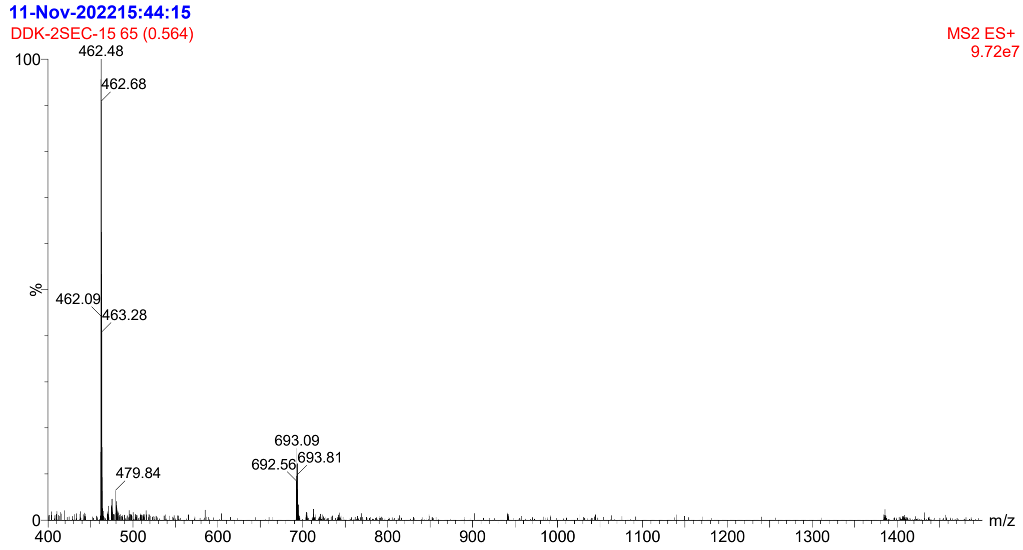

(b)

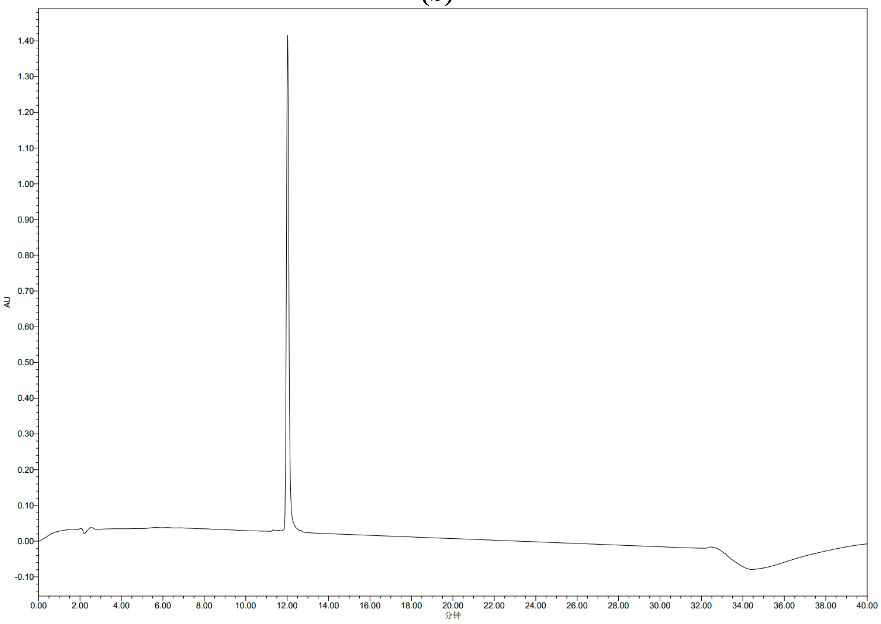

(c)

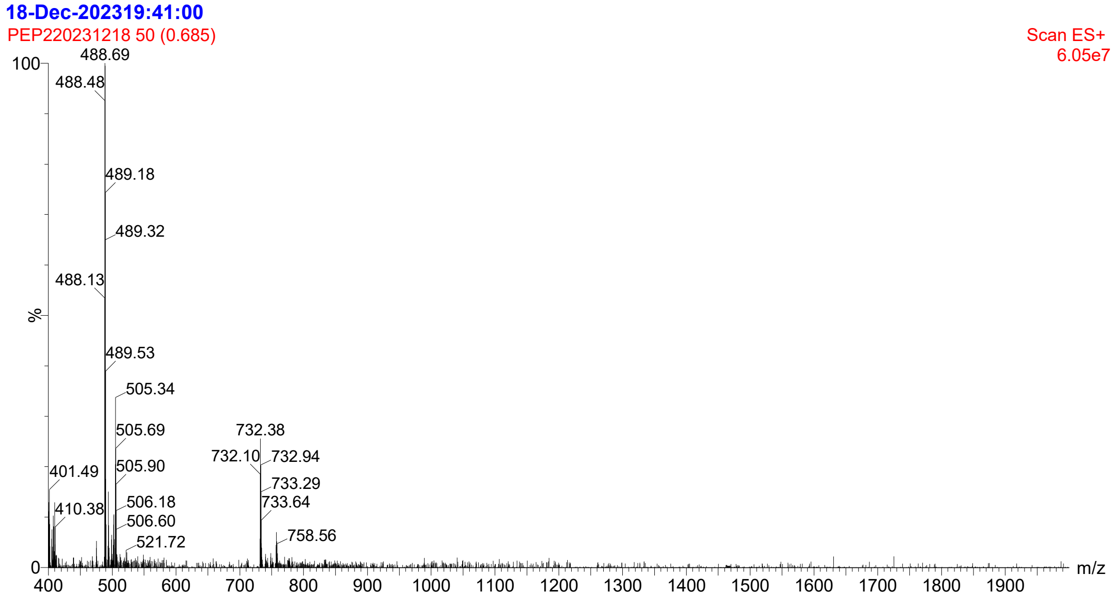

18-Dec-202319:41:00
PEP220231218 50 (0.685)
Scan ES+
6.05e7
100
%
0
488.69
488.48
489.18
489.32
488.13
489.53
505.34
505.69
505.90
506.18
506.60
521.72
401.49
410.38
732.38
732.10
732.94
733.29
733.64
758.56
m/z
400
500
600
700
800
900
1000
1100
1200
1300
1400
1500
1600
1700
1800
1900

(d)

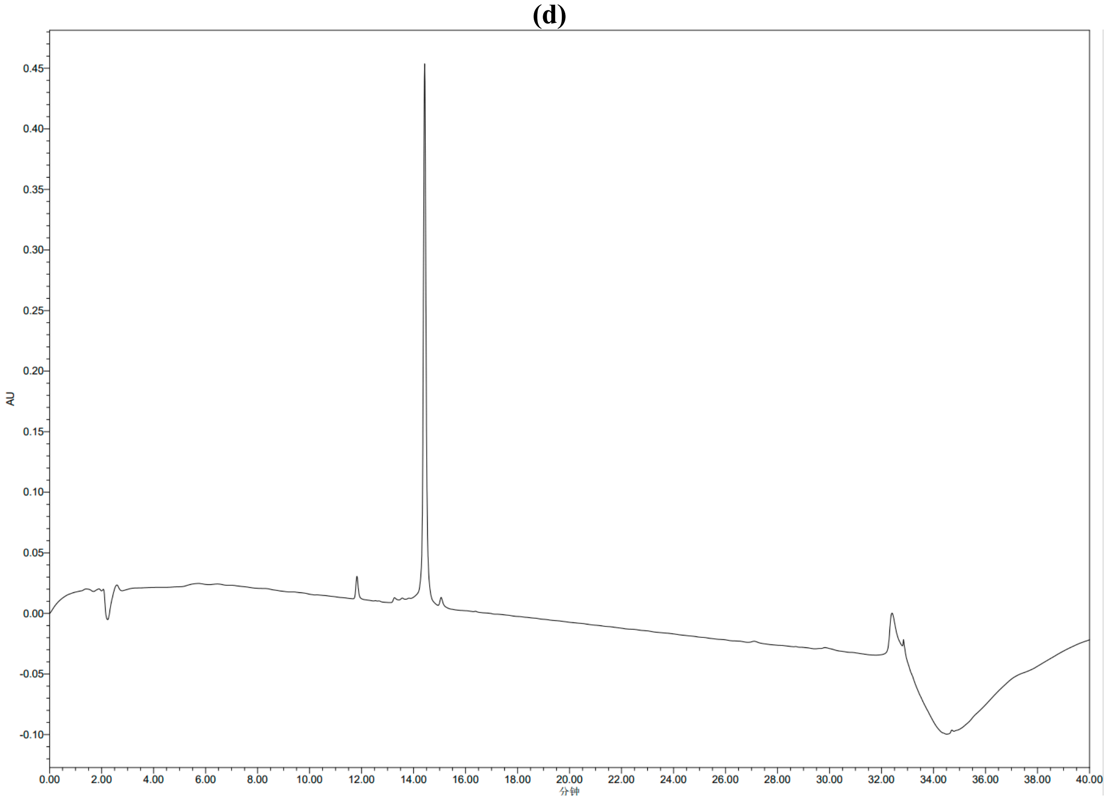

AU
0.45
0.40
0.35
0.30
0.25
0.20
0.15
0.10
0.05
0.00
-0.05
-0.10
0.00
2.00
4.00
6.00
8.00
10.00
12.00
14.00
16.00
18.00
20.00
22.00
24.00
26.00
28.00
30.00
32.00
34.00
36.00
38.00
40.00
分钟

(e)

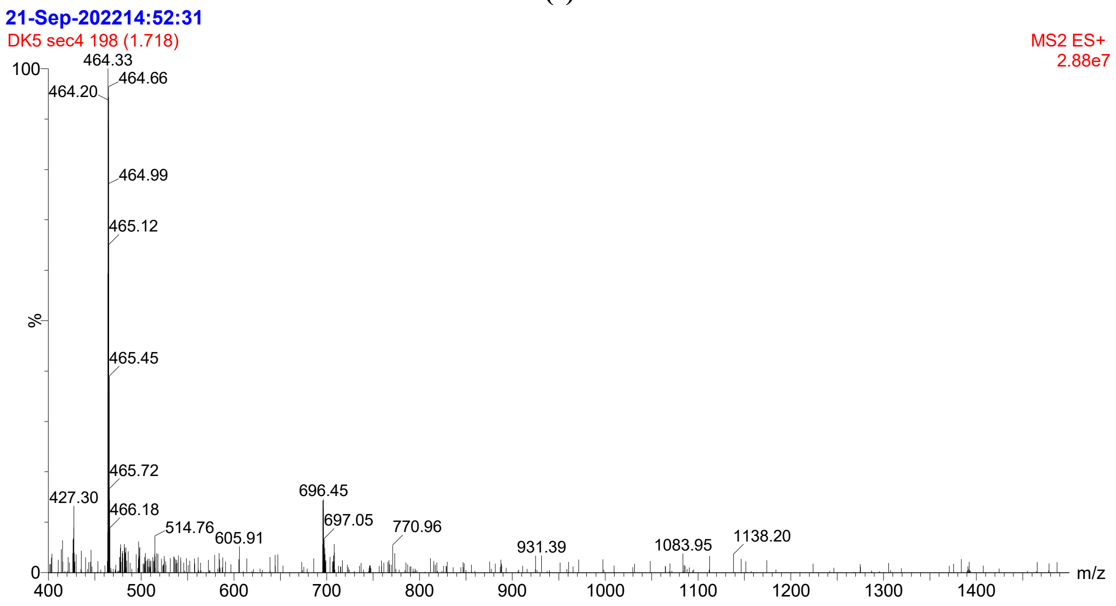
21-Sep-202214:52:31
DK5 sec4 198 (1.718)
MS2 ES+
2.88e7
100
%
0
464.33
464.20
464.66
464.99
465.12
465.45
465.72
466.18
427.30
514.76
605.91
696.45
697.05
770.96
931.39
1083.95
1138.20
400
500
600
700
800
900
1000
1100
1200
1300
1400
m/z

(f)

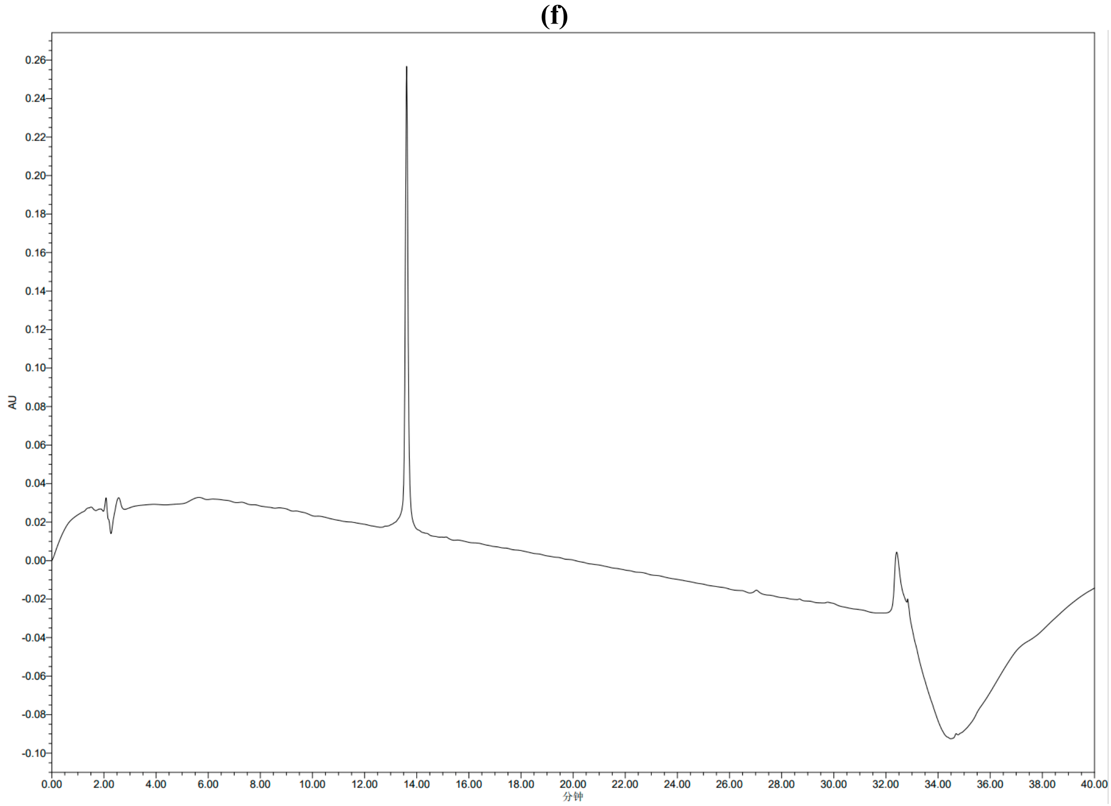
AU
0.26
0.24
0.22
0.20
0.18
0.16
0.14
0.12
0.10
0.08
0.06
0.04
0.02
0.00
-0.02
-0.04
-0.06
-0.08
-0.10
0.00
2.00
4.00
6.00
8.00
10.00
12.00
14.00
16.00
18.00
20.00
22.00
24.00
26.00
28.00
30.00
32.00
34.00
36.00
38.00
40.00
分钟

(g)

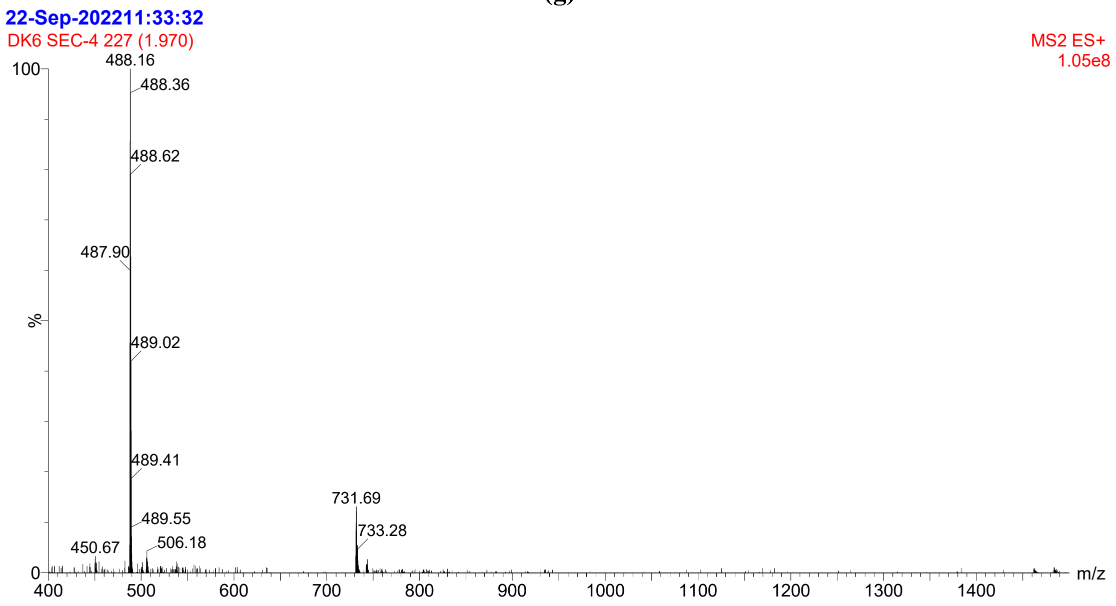

22-Sep-202211:33:32
DK6 SEC-4 227 (1.970)
MS2 ES+
1.05e8
488.16
488.36
488.62
487.90
489.02
489.41
489.55
506.18
450.67
731.69
733.28
%
m/z
100
0
400
500
600
700
800
900
1000
1100
1200
1300
1400

(h)

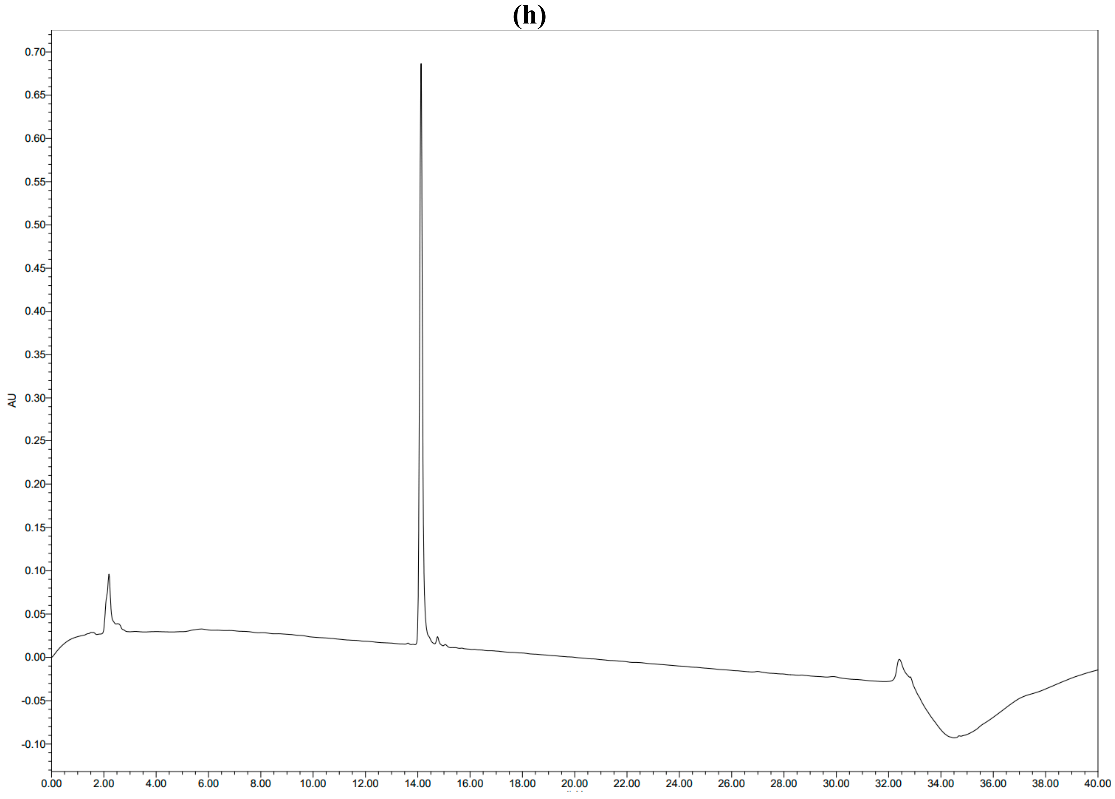

AU
0.70
0.65
0.60
0.55
0.50
0.45
0.40
0.35
0.30
0.25
0.20
0.15
0.10
0.05
0.00
-0.05
-0.10
0.00
2.00
4.00
6.00
8.00
10.00
12.00
14.00
16.00
18.00
20.00
22.00
24.00
26.00
28.00
30.00
32.00
34.00
36.00
38.00
40.00

(i)

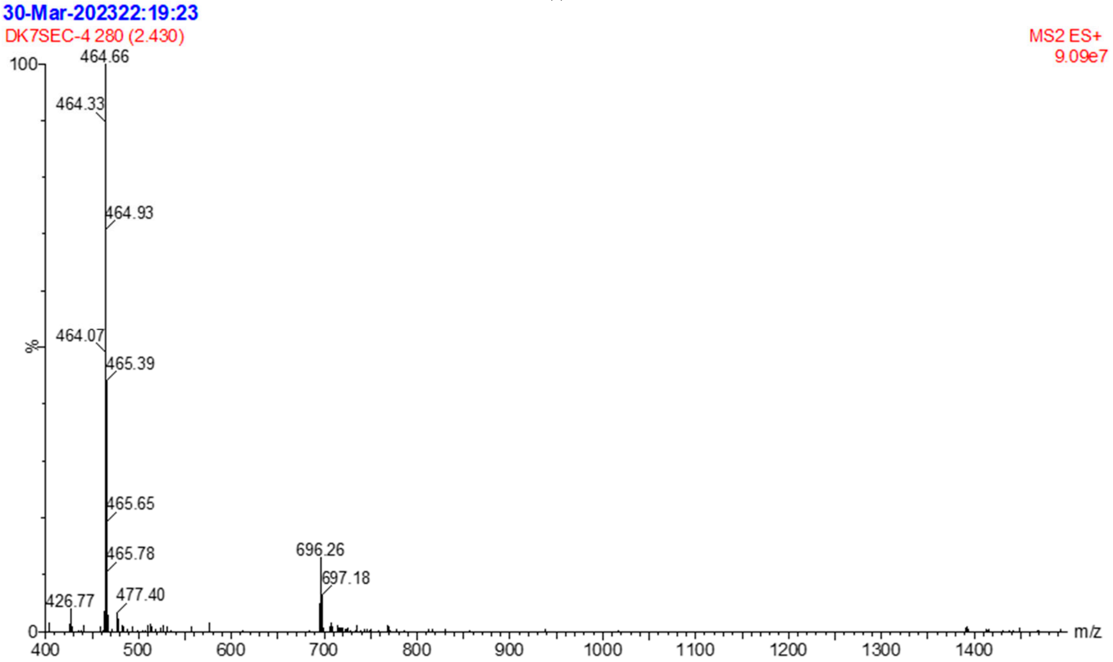
30-Mar-202322:19:23
DK7SEC-4 280 (2.430)
MS2 ES+
9.09e7
100
%
0
464.66
464.33
464.93
464.07
465.39
465.65
465.78
426.77
477.40
696.26
697.18
400
500
600
700
800
900
1000
1100
1200
1300
1400
m/z

(j)

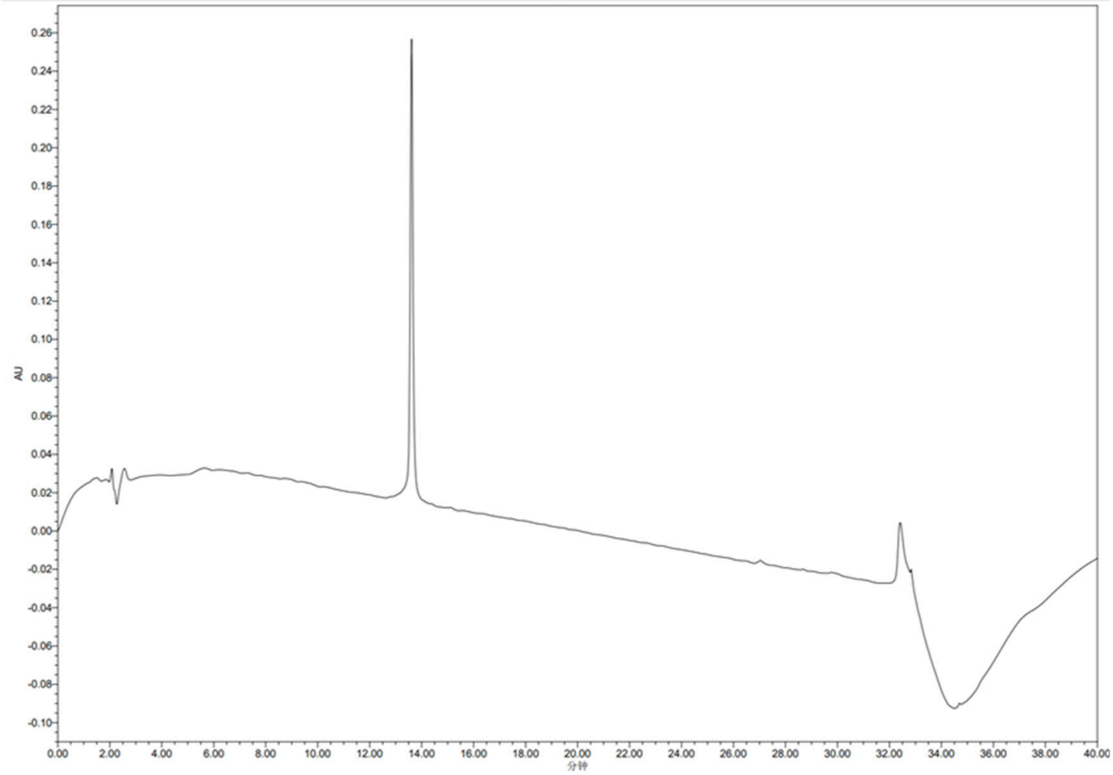
AU
分钟

(k)

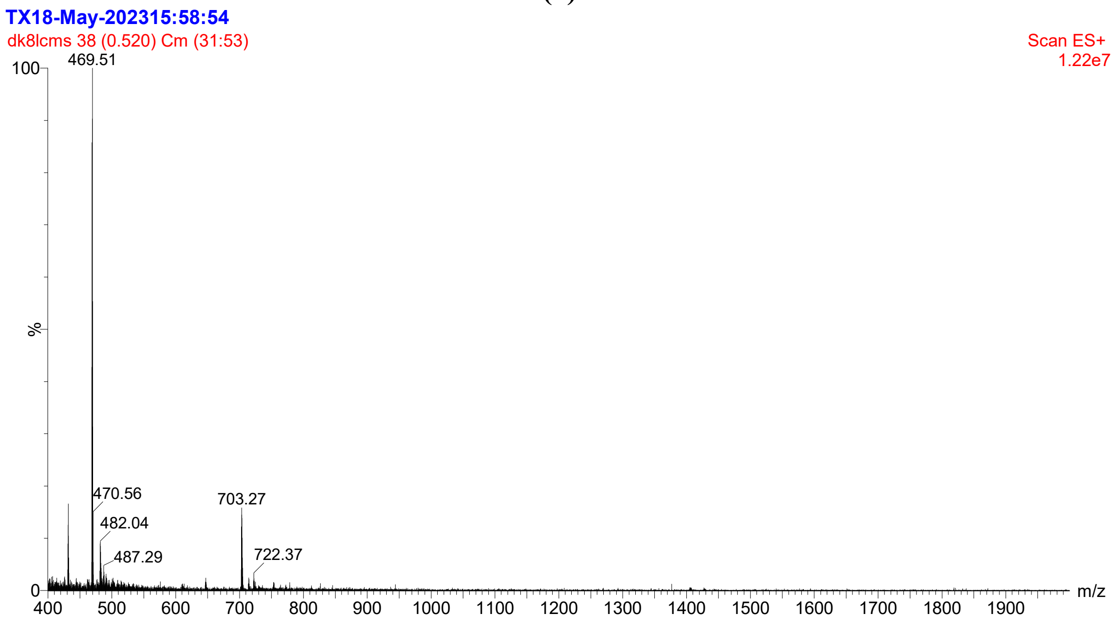
TX18-May-202315:58:54
dk8lcms 38 (0.520) Cm (31:53)
Scan ES+
1.22e7
469.51
470.56
482.04
487.29
703.27
722.37
%
m/z
100
0
400
500
600
700
800
900
1000
1100
1200
1300
1400
1500
1600
1700
1800
1900

(l)

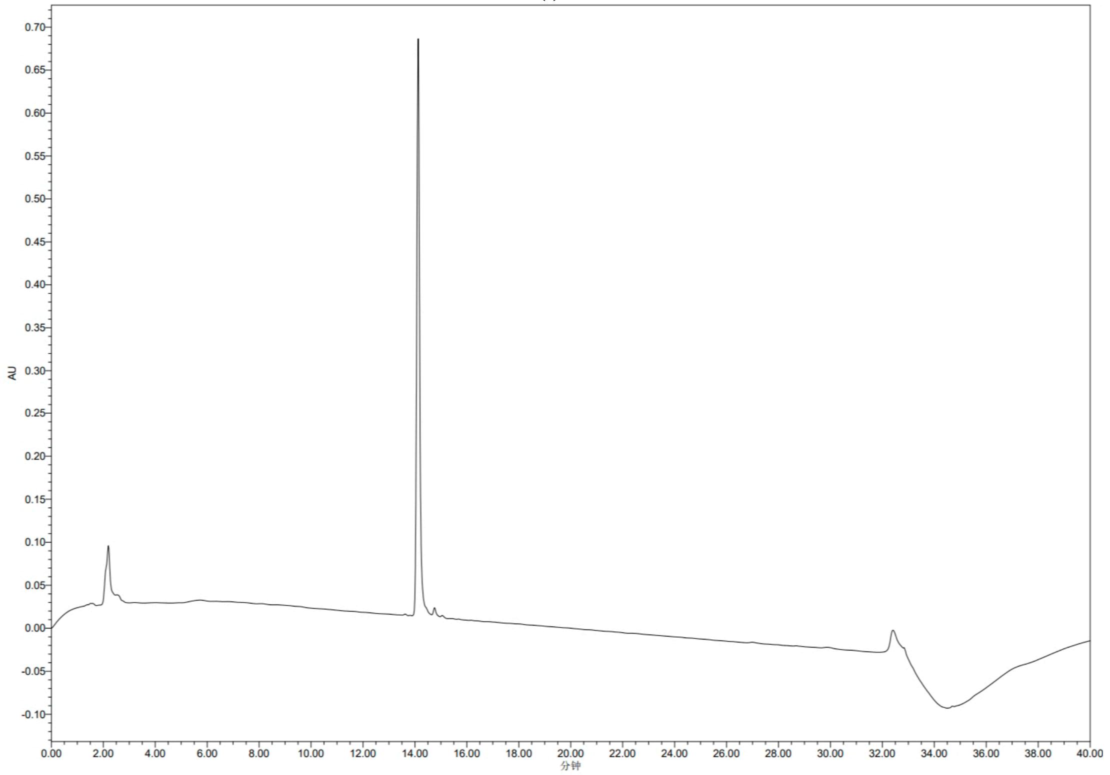
AU
分钟
0.70
0.65
0.60
0.55
0.50
0.45
0.40
0.35
0.30
0.25
0.20
0.15
0.10
0.05
0.00
-0.05
-0.10
0.00
2.00
4.00
6.00
8.00
10.00
12.00
14.00
16.00
18.00
20.00
22.00
24.00
26.00
28.00
30.00
32.00
34.00
36.00
38.00
40.00

(m)

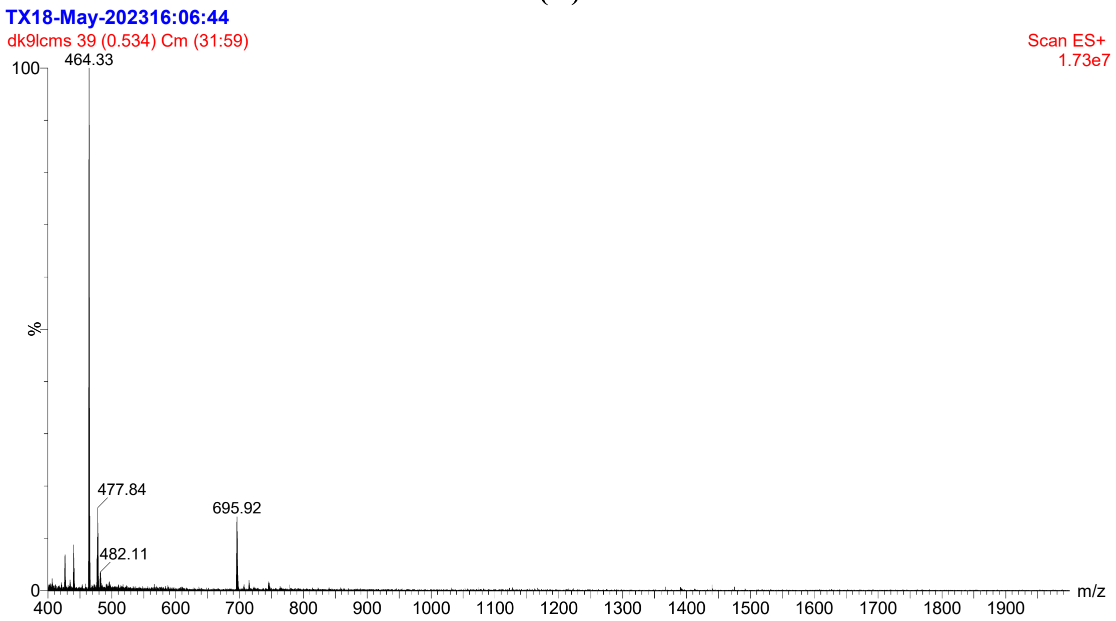
TX18-May-202316:06:44
dk9lcms 39 (0.534) Cm (31:59)
Scan ES+
1.73e7
464.33
477.84
482.11
695.92
100
%
0
m/z
400
500
600
700
800
900
1000
1100
1200
1300
1400
1500
1600
1700
1800
1900

(n)

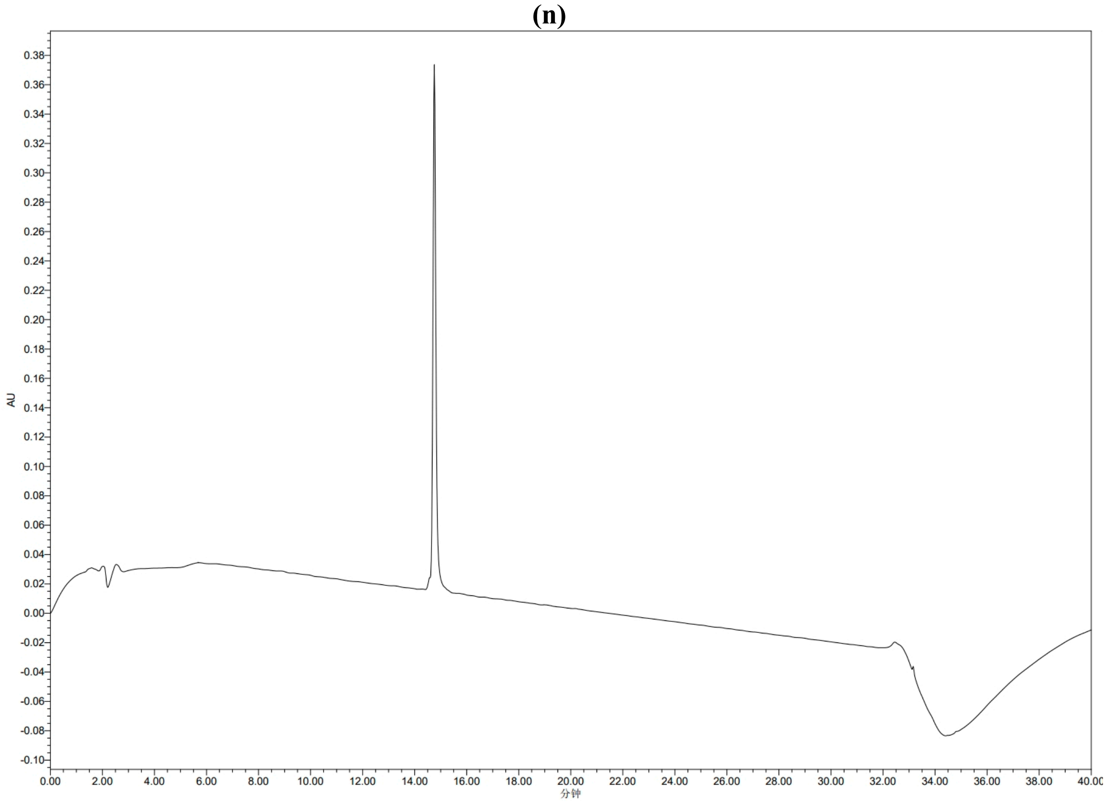
AU
分钟

(o)

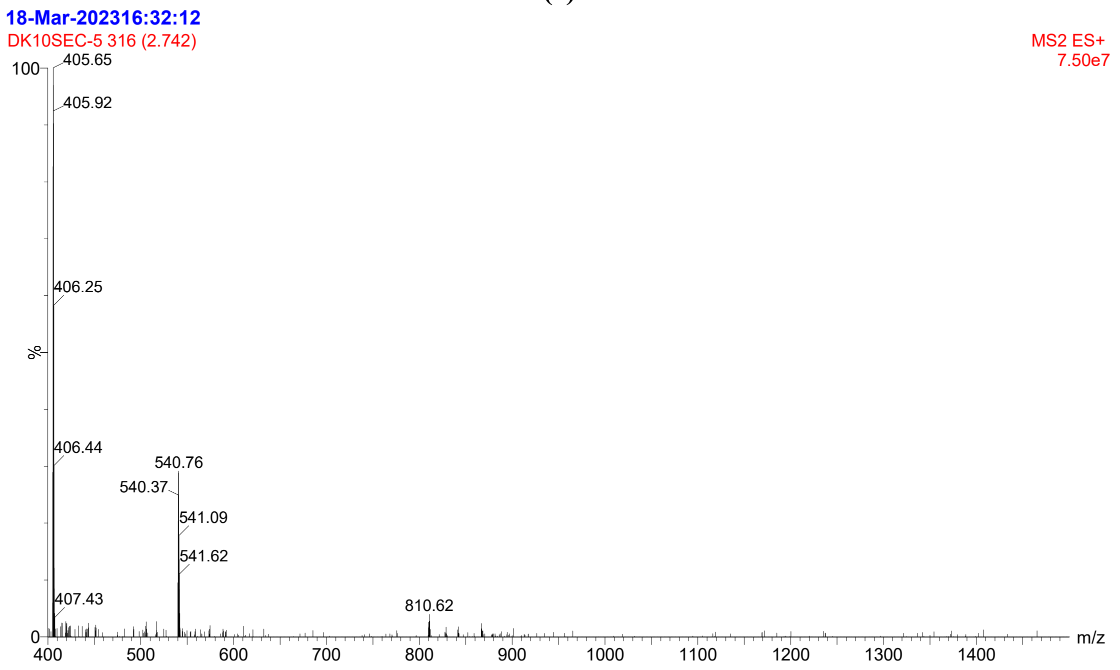
18-Mar-202316:32:12
DK10SEC-5 316 (2.742)
MS2 ES+
7.50e7
405.65
405.92
406.25
406.44
540.76
540.37
541.09
541.62
407.43
810.62
%
m/z

(p)

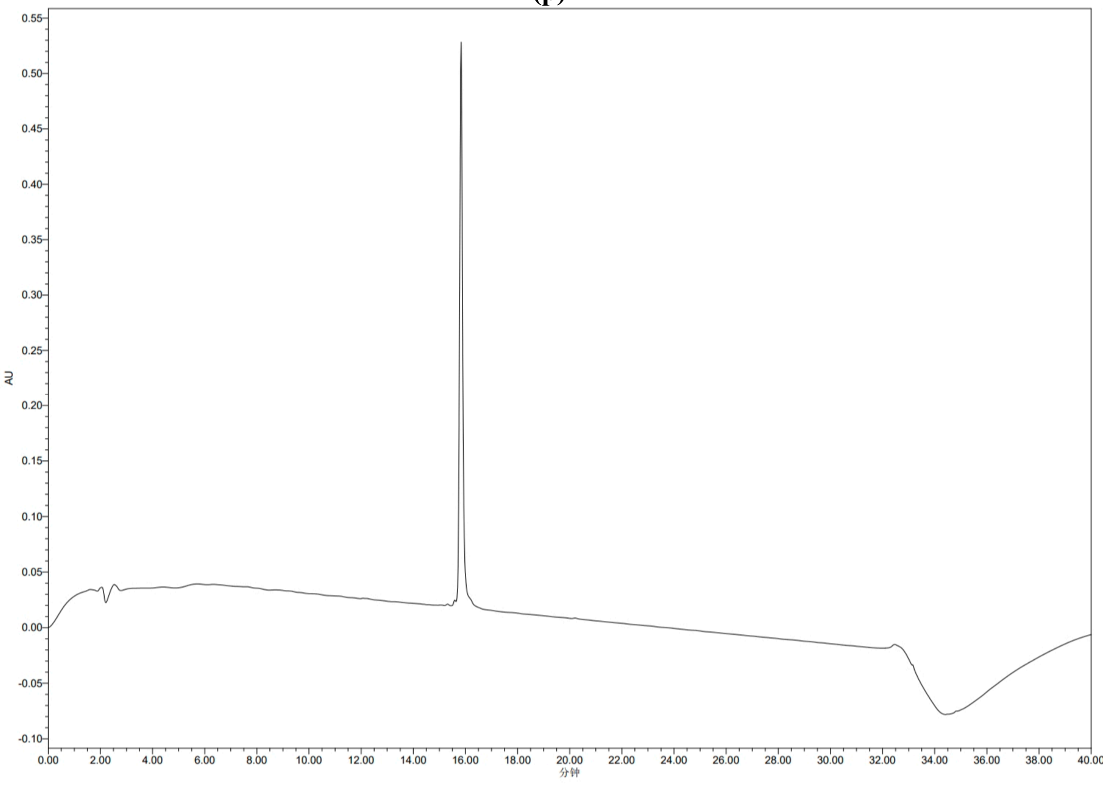
AU
分钟

(q)

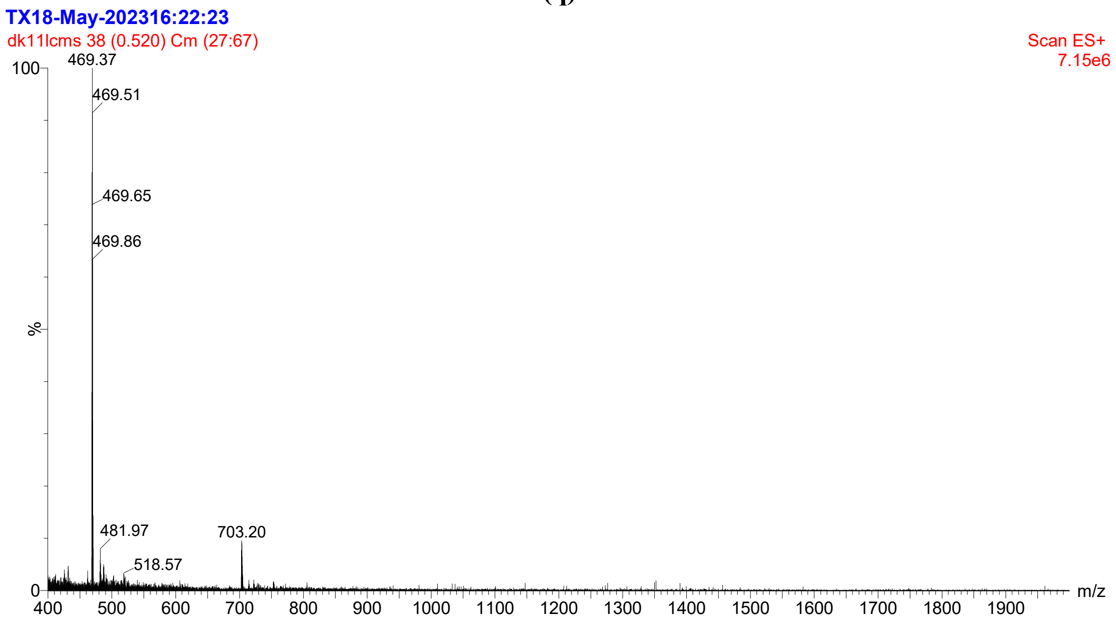

(r)

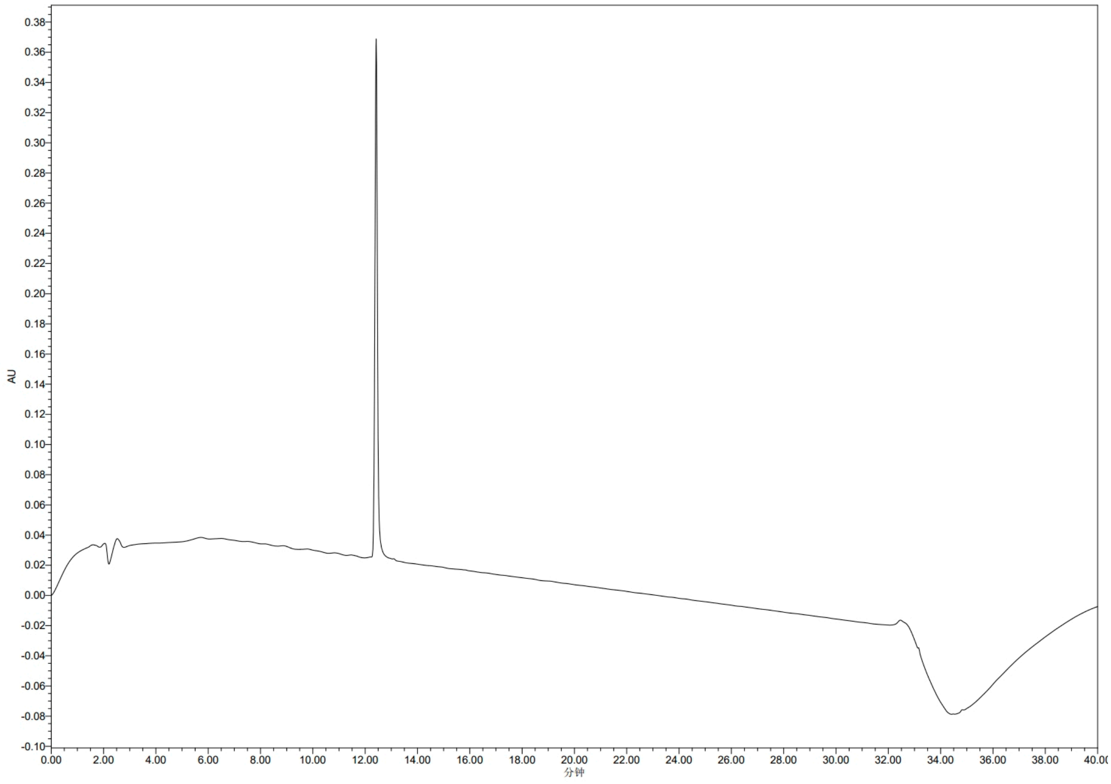

Figure S1. (**a**) LC-MS analysis of Pep 1. (**b**) HPLC analysis of the Pep 1. (**c**) LC-MS analysis of Pep 2. (**d**) HPLC analysis of the Pep 2. (**e**) LC-MS analysis of Pep 3. (**f**) HPLC analysis of the Pep 3. (**g**) LC-MS analysis of Pep 4. (**h**) HPLC analysis of the Pep 4. (**i**) LC-MS analysis of Pep 5. (**j**) HPLC analysis of the Pep 5. (**k**) LC-MS analysis of Pep 6. (**l**) HPLC analysis of the Pep 6. (**m**) LC-MS analysis of Pep 7. (**n**) HPLC analysis of the Pep 7. (**o**) LC-MS analysis of Pep 8. (**p**) HPLC analysis of the Pep 8. (**q**) LC-MS analysis of Pep 9. (**r**) HPLC analysis of the Pep 9.
